# Cytomegalovirus Proctitis Mimicking Inflammatory Bowel Disease in an Immunocompetent Elderly Patient: A Diagnostic Challenge

**DOI:** 10.7759/cureus.86042

**Published:** 2025-06-15

**Authors:** Rachael Hagen, Teresa Da Cunha, Alexander Potashinsky

**Affiliations:** 1 Internal Medicine, University of Connecticut, Farmington, USA; 2 Gastroenterology and Hepatology, University of Connecticut, Hartford, USA; 3 Gastroenterology and Hepatology, Hospital of Central Connecticut, New Britain, USA

**Keywords:** cmv proctitis, colitis, cytomegalovirus, immunocompetent, proctitis

## Abstract

Cytomegalovirus (CMV) colitis is an opportunistic virus typically diagnosed in immunocompromised patients. CMV proctitis is rarely reported in immunocompetent hosts. We describe a 71-year-old immunocompetent male who presented with persistent diarrhea and was CMV-negative on initial histopathology staining but was found to have severe CMV proctitis with rectal involvement diagnosed following repeat flexible sigmoidoscopy. CMV proctitis is likely an underrecognized cause of bloody diarrhea in the elderly. Early consideration of CMV proctitis in immunocompetent elderly patients with refractory colitis may prevent prolonged morbidity and unnecessary immunosuppressive therapy.

## Introduction

Cytomegalovirus (CMV) is prevalent among immunocompromised patients and can cause infections affecting various organs. Immunocompetent hosts are rarely affected and may develop self-limiting, mononucleosis-like symptoms [1]. CMV colitis presents with symptoms such as rectal pain and hematochezia [2]. Following primary CMV infection, the virus remains latent, with the potential for reactivation in the colon of immunocompromised individuals, which can cause gastrointestinal inflammation or hemorrhage, leukopenia, thrombocytopenia, and lymphocytosis [3,4]. In contrast, CMV colitis in immunocompetent patients is more commonly associated with primary infection. The prevalence of CMV proctitis in immunocompetent individuals is rare and varies among subgroups (0.3-31%) [5]. One study reported 51 immunocompetent patients at a tertiary care university hospital in a 19-year time period [6]. Rectal involvement is referred to as CMV proctitis and is particularly uncommon compared to CMV colitis, when CMV affects the colon only and accounts for 94% of gastrointestinal CMV cases [7,8]. Here, we describe a prolonged case of CMV proctosigmoiditis in an immunocompetent patient. 

CMV can lead to severe morbidity in immunocompromised patients, with increasing recognition of complications in select immunocompetent individuals [8]. Risk factors for CMV involve immunosuppressive states, such as Acquired Immune Deficiency Syndrome (AIDS), older age, critical illness, steroid use, and comorbidities such as diabetes [9]. CMV colitis in immunocompetent patients remains a poorly understood yet increasingly recognized cause of colitis. This case adds to the growing body of evidence suggesting that CMV should be included in the differential diagnosis for colitis in older, hospitalized patients.

Despite the use of immunohistochemical (IHC) staining as the gold standard for diagnosis, false negatives can occur due to sampling error or low viral loads in early infection stages [7,10]. This highlights the importance of repeat biopsies when initial findings are inconclusive. While antiviral therapy is generally reserved for immunocompromised patients, emerging literature suggests that select immunocompetent individuals, particularly elderly males with diabetes or renal disease, may benefit from treatment with ganciclovir or valganciclovir [1,7]. This case underscores the diagnostic challenges of CMV colitis and reinforces the need for clinicians to maintain a high index of suspicion, particularly in older patients with persistent colitis refractory to standard therapy. This article was previously presented as a meeting abstract at the 2024 American College of Gastroenterology Annual Meeting on October 27, 2024 in Philadelphia, PA

## Case presentation

A 71-year-old male with a history of diabetes presented with two days of hematochezia and abdominal pain. Stool testing was positive for both rotavirus and norovirus, which are typically self-limiting and rarely associated with significant lower gastrointestinal bleeding. Despite this, he was prescribed a 10-day course of amoxicillin for unclear reasons. Three days later, he returned with persistent rectal bleeding and severe left lower quadrant pain. He was afebrile without leukocytosis. Laboratory findings revealed a high normal erythrocyte sedimentation rate, an elevated high-sensitivity C-reactive protein, and an elevated fecal calprotectin, suggesting an inflammatory process in the colon (Table 1). 

**Table 1:** Initial laboratory results Initial laboratory results were remarkable for elevated inflammatory markers, such as fecal calprotectin, ESR, and CRP. Of note, CMV PCR levels were not tested until one week after the initial presentation and were positive. Five weeks later, his CMV levels became undetectable after antiviral treatment. WBC: white blood cell, Hb: hemoglobin, Hct: hematocrit, BUN: blood urea nitrogen, AST: aspartate aminotransferase, ALT: alanine transaminase, ALP: alkaline phosphatase, ESR: erythrocyte sedimentation rate, CRP: C-reactive protein, CMV: cytomegalovirus, RPR: rapid plasma reagin, HIV: human immunodeficiency virus, HSV: herpes simplex virus, PCR: polymerase chain reaction, Ig: Immunoglobulin, Ab: antibody.

Tests	Results	Results one Week Later	Results Five Weeks Later	Reference Range
Complete Blood Counts				
WBC	6	6.7	5	4.0-11.0 thou/uL
Hb	12.2	11	11.8	13-18 (g/dL)
Hct	37.7	33.9	36	35-46%
Platelet count	126	162	170	150-400 x109/L
Basic Metabolic Panel				
Sodium	137	135	138	135-145 mmol/L
Potassium	4.2	3.8	4	3.5-5 mmol/L
Chloride	104	100	101	98-107 mmol/L
BUN	15	7	10	10-50 mg/dl
Creatinine	1.4	0.8	0.9	0.5-1.3 mg/dl
Calcium	8.7	8.3	8.5	8.5-10.5 mg/dl
Liver Function Tests				
Total Bilirubin	0.6	0.5	0.5	0.3-1.0 mg/dL
Direct Bilirubin	<0.2	<0.2	<0.2	<0.3 mg/dL
AST	36	32	29	13-39 U/L
ALT	16	17	15	7-52 U/L
ALP	77	73	70	34-104 U/L
Lipase	42	NA	NA	13-60 U/L
ESR	20	NA	NA	<20 mm/hr
CRP	1.94	NA	NA	<1.0 mg/dL
Fecal calprotectin	>8000 mcg/g	NA	NA	<80 mcg/g
*Clostridium difficile* toxin and NAP1 PCR	Negative	NA	NA	Negative
CMV PCR (log10)	NA	2.76	<1.72	<1.72 log10
CMV PCR (IU/mL)	NA	572	<53	<53 IU/mL
CMV IgM	NA	<0.2	NA	<0.2
Gonorrhea	NA	Negative	NA	Negative
RPR	NA	Negative	NA	Negative
Chlamydia	NA	Negative	NA	Negative
Entamoeba histolytica	NA	Negative	NA	Negative
HIV 1/2	NA	Negative	NA	Negative
HSV 1 Ab Screen, IgM	NA	Negative	NA	Negative
HSV 2 Ab Screen, IgM	NA	Negative	NA	Negative
HSV-2 Antibody IgG	NA	Negative	NA	Negative

**Figure 1:**
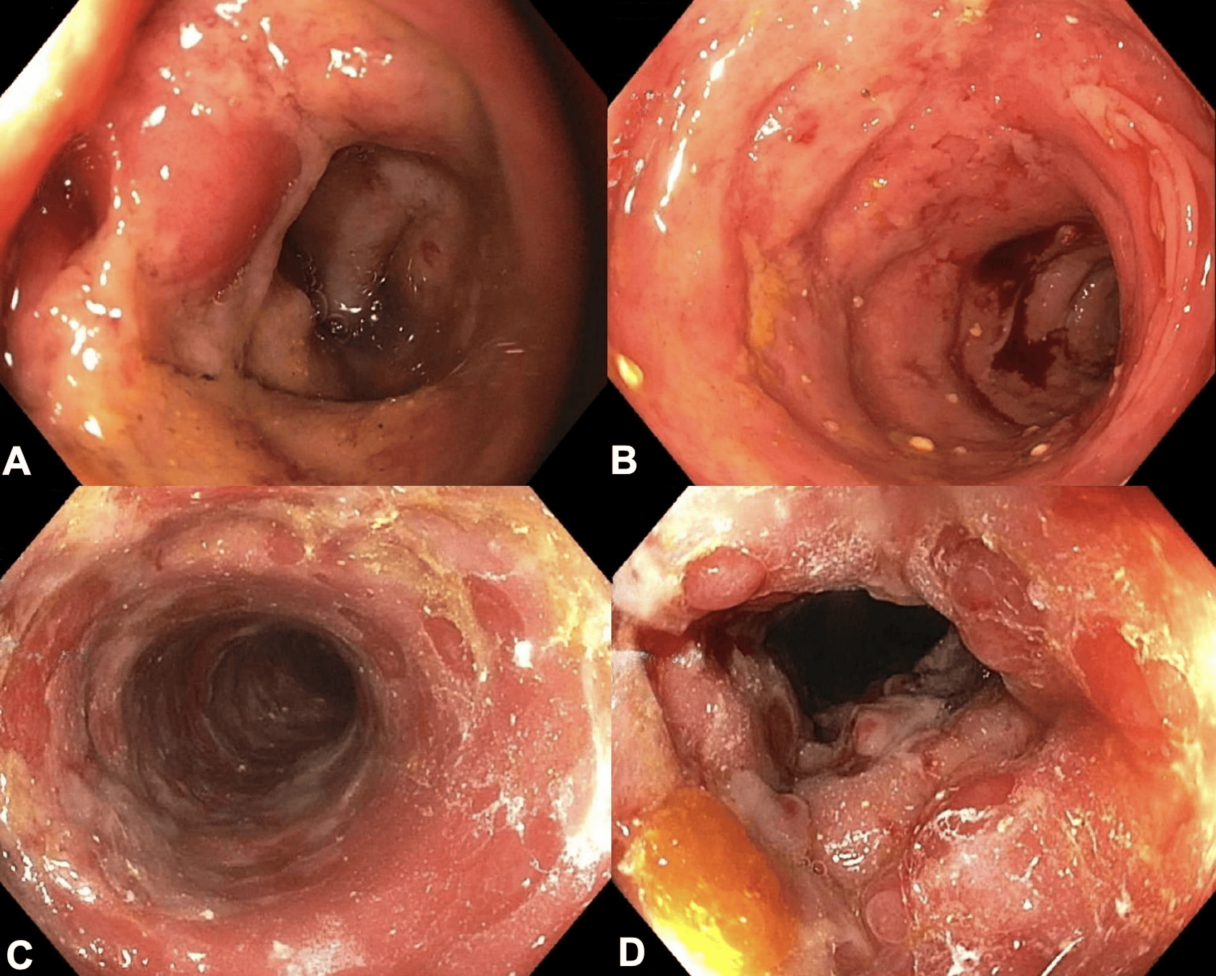
Sigmoidoscopy images of the colon A-B. Initial flexible sigmoidoscopy demonstrated erythematous and friable mucosa with contact bleeding in the rectum and sigmoid colon. C-D. A week later, flexible sigmoidoscopy visualized more severe congestion with hemorrhagic, inflamed, and ulcerated mucosa in the rectum, sigmoid, and descending colon. These findings were clinically correlated with worsening symptoms, including increased frequency of diarrhea, rectal bleeding, and pain.

*Clostridium difficile* testing was negative. Abdominal computed tomography (CT) demonstrated sigmoid and rectal thickening, raising concern for ischemic colitis, inflammatory bowel disease, or an atypical viral infection. Flexible sigmoidoscopy performed on his sixth day of experiencing symptoms showed congested, erythematous, and friable mucosa with bleeding in the rectum and sigmoid colon (Figures 1A, 1B). These findings were nonspecific but concerning for severe colitis. Initial biopsies revealed active colitis with ulceration and acute inflammation. Immunohistochemical (IHC) staining for CMV was negative at this stage.

Despite treatment with antibiotics, budesonide, and mesalamine, the patient’s bloody diarrhea persisted, suggesting an alternative or superimposed pathology beyond viral gastroenteritis. He denied a history of anal intercourse. Given the continued progression of symptoms 12 days after symptom onset, a repeat CT scan of the abdomen and pelvis showed worsening proctosigmoiditis. Flexible sigmoidoscopy performed on his fourteenth day of symptoms revealed severe congestion with hemorrhagic and ulcerated mucosa (Figures 1C, 1D).

**Figure 2:**
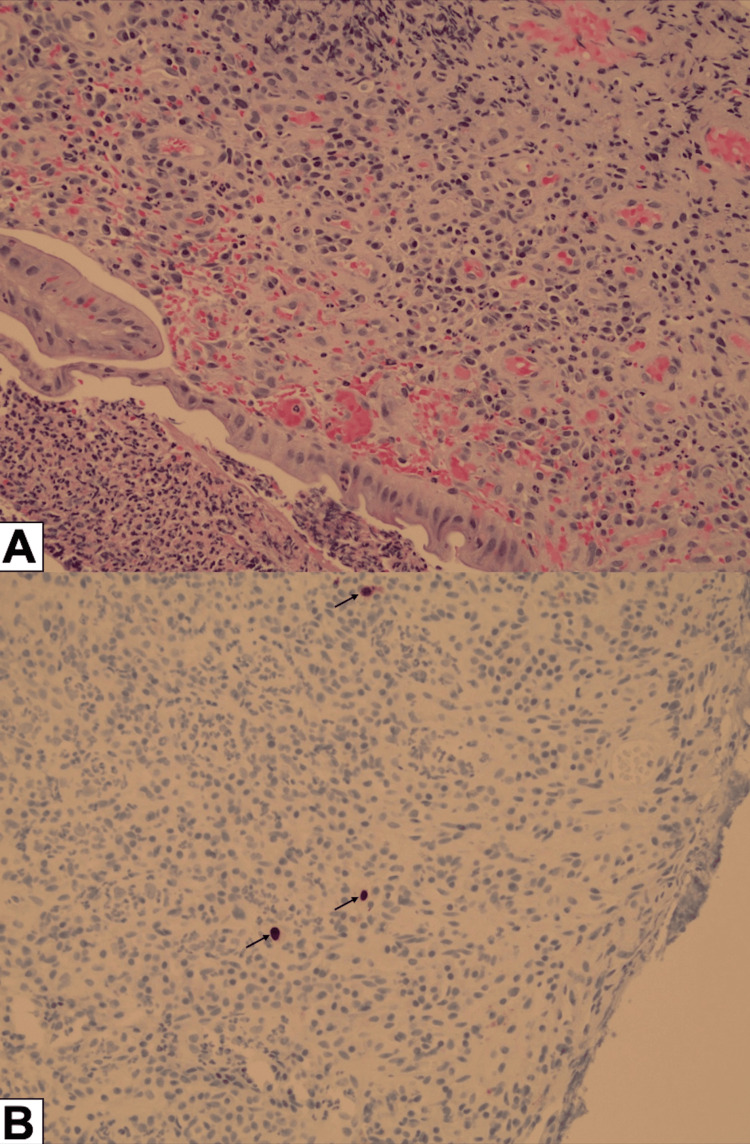
Immunohistochemistry results A. Hematoxylin and eosin staining (200X) revealed ulcerated and acutely inflamed mucosa with granulation tissue and focal glandular atypia. B. Immunohistochemistry staining with monoclonal antibodies for cytomegalovirus (200X) showed positive uptake for inclusion bodies (black arrows).

Repeat biopsies showed ulcerated mucosa (Figure 2A), and IHC staining now indicated rare CMV-positive cells with CMV intranuclear inclusions (Figure 2B). CMV serologies were positive, demonstrating an elevated CMV polymerase chain reaction (PCR), confirming CMV proctitis as a contributing factor to the patient’s symptoms. Testing for sexually transmitted infections was performed to exclude risk factors for CMV colitis and was negative.

Although the treatment of CMV colitis in immunocompetent individuals remains controversial, the decision was made to treat the patient given the concern for concomitant inflammatory bowel disease (IBD) and the severity of symptoms. He was treated with intravenous ganciclovir with initial improvement, which was transitioned to oral valganciclovir at discharge per infectious disease recommendations. After three weeks of treatment, he reported ongoing excessive diarrhea with bowel movements occurring up to ten times daily associated with occasional fecal incontinence. The decision for longer treatment was requested by the patient due to ongoing symptoms and was not due to CMV viral load. After an additional two weeks of treatment, he experienced symptom resolution with CMV viral load becoming undetectable.

## Discussion

Cytomegalovirus (CMV) colitis is typically associated with immunocompromised states, making its occurrence in immunocompetent individuals an unusual and diagnostically challenging entity [8]. Initial biopsies were negative for CMV, which contributed to a prolonged 22-day hospitalization. He was slow to respond to treatment, requiring a total of five weeks of induction therapy, exceeding the standard two to three weeks typically recommended [11]. This case highlights the importance of maintaining a high index of suspicion when initial colonoscopy findings are not revealing and emphasizes the value of performing repeat flexible sigmoidoscopy with additional biopsies for accurate diagnosis. 

Most cases occur in elderly patients with diabetes and other comorbidities [2,12]. Typically, CMV proctitis develops following hospitalization, as reported in 72.7% of cases in one review [7]. This suggests that CMV proctitis should be more widely recognized as a potential cause of diarrhea in older, immunocompetent hospitalized patients. A literature review identified the most common symptoms of CMV colitis in immunocompetent patients as diarrhea (76%), abdominal pain (52%), and hematochezia (27%) [10]. Diagnostic workup includes ruling out common causes of colitis, including *Clostridium difficile*, sexually transmitted infections, ischemic colitis, human immunodeficiency virus (HIV), and performing a colonoscopy to exclude IBD. 

Obtaining colonic biopsies in patients with persistent colitis is important to rule out an acute CMV infection. The gold standard for diagnosis is CMV PCR and IHC staining of colonic biopsies using CMV-specific monoclonal antibodies. Importantly, IHC staining must be specifically requested by endoscopists and is not routinely performed. In this case, initial biopsies demonstrated acute colitis with ulceration, but CMV IHC was negative, potentially due to early-stage infection, where CMV levels may be below the threshold of detection on IHC stain, or sampling error, given the patchy involvement of CMV in the colon. Over time, the viral load can increase, making CMV detectable on repeat testing. To enhance diagnostic yield, multiple biopsies should be taken from different sites, especially areas with endoscopic evidence of inflammation. Diagnostic techniques such as IHC, in situ hybridization, testing multiple tissue blocks, and quantitative PCR can improve detection rates [13,14]. A limitation of this case was the delayed testing of CMV PCR, which contributed to a prolonged time to diagnosis.

Histopathological findings such as “owl’s eye” inclusion bodies are also highly specific [3]. Endoscopic features, including well-defined, punched-out ulcerations and pseudomembrane formation, are observed in 70-80% of patients with CMV colitis [1,8]. In a review of CMV colitis in immunocompetent patients, rectal ulcers were seen in 85.0% of cases, polypoidal masses in 30.0%, and rectal fistulas or sinus tracts in 10.0% [8]. These findings emphasize the importance of thorough endoscopic evaluation and repeat biopsies in cases with inconclusive initial results.

Serological testing for CMV immunoglobulin (Ig)M and IgG levels is not diagnostic for CMV colitis but can indicate an acute systemic infection, reactivation, or prior exposure [1]. The CMV antigenemia assay can aid in early diagnosis but has a low sensitivity [1]. Real-time PCR CMV DNA quantification is more sensitive for active infection than serologies or IHC and can complement endoscopic findings for diagnosis [14,15].

Antiviral therapy is rarely indicated in immunocompetent patients, as there is no evidence of significant benefit and the potential for side effects. However, treatment may be considered in select cases, such as patients over the age of 55 with comorbidities (e.g., renal failure or diabetes) that may impair immunity, systemic involvement, severe or persistent symptoms despite supportive care, and endoscopic or histologic evidence of severe disease (e.g., deep tissue invasion, extensive ulcerations) [1]. Treatment response is typically monitored clinically by a reduction in diarrhea, abdominal pain, and rectal bleeding. In severe cases with persistent symptoms, endoscopic reevaluation may be warranted to assess for mucosal healing. Routine monitoring of CMV PCR and inflammatory markers, such as C-reactive protein and fecal calprotectin, is not routinely performed but can be beneficial as declining levels may help indicate treatment efficacy. 

In this case, given concerns for possible IBD and severe symptoms, the decision was made to initiate antiviral therapy led by the infectious disease team. Many case reports of CMV colitis in immunocompetent patients describe successful treatment with ganciclovir, which remains the gold standard at 5 mg/kg twice daily for two to three weeks, with a transition to oral valganciclovir for outpatient management [16]. However, the reason for its preference over valganciclovir remains unclear [2,8]. Oral valganciclovir, a prodrug of ganciclovir, has demonstrated similar efficacy with better tolerability without the risk of pancytopenia [17].

CMV should be considered as a potential cause of bloody diarrhea in patients with risk factors, such as older age and diabetes, after common conditions have been excluded. The non-specific presentation can lead to underdiagnosis, which may be attributed to other conditions, especially in the elderly [7]. Elderly patients tend to be affected by CMV colitis due to immune senescence, age-related decline in immunity, and comorbidities [18]. Additionally, endoscopists must have a suspicion of CMV colitis due to the specific histological stains that are required. This case emphasizes the importance of repeating colonic biopsies when initial pathology is unrevealing to determine the underlying cause of colonic ulcerations. Severe cases may require an extended treatment course for full eradication.

## Conclusions

This case underscores the diagnostic challenges of CMV proctocolitis in immunocompetent individuals and highlights the importance of including it in the differential diagnosis for elderly diabetic patients with persistent gastrointestinal symptoms. Repeat endoscopy should be considered in cases of refractory colitis, as initial negative IHC staining for CMV may reflect sampling error or early-stage infection. This reinforces the need for additional biopsies when clinical suspicion remains high. Moreover, the patient’s prolonged course suggests that prolonged antiviral therapy beyond the standard duration may be necessary for symptom resolution in select cases with ongoing gastrointestinal symptoms. Given the increasing recognition of CMV colitis in immunocompetent patients, clinicians should consider it as part of the differential diagnosis for refractory colitis, particularly in hospitalized individuals with persistent colitis unresponsive to conventional therapy. Further research is warranted to better understand this condition, which rarely affects immunocompetent hosts.
